# Conserved antigenic sites between MERS-CoV and Bat-coronavirus are revealed through sequence analysis

**DOI:** 10.1186/s13029-016-0049-7

**Published:** 2016-03-09

**Authors:** Refat Sharmin, Abul B. M. M. K. Islam

**Affiliations:** Research and Development Department, Incepta Vaccine Ltd., Zirabo, Savar, Dhaka 1341 Bangladesh; Department of Genetic Engineering and Biotechnology, University of Dhaka, Science Complex Building, Dhaka, 1000 Bangladesh

**Keywords:** MERS-CoV, HKU4, HKU5, Epitope

## Abstract

**Background:**

MERS-CoV is a newly emerged human coronavirus reported closely related with HKU4 and HKU5 Bat coronaviruses. Bat and MERS corona-viruses are structurally related. Therefore, it is of interest to estimate the degree of conserved antigenic sites among them. It is of importance to elucidate the shared antigenic-sites and extent of conservation between them to understand the evolutionary dynamics of MERS-CoV.

**Results:**

Multiple sequence alignment of the spike (S), membrane (M), enveloped (E) and nucleocapsid (N) proteins was employed to identify the sequence conservation among MERS and Bat (HKU4, HKU5) coronaviruses. We used various *in silico* tools to predict the conserved antigenic sites. We found that MERS-CoV shared 30 % of its S protein antigenic sites with HKU4 and 70 % with HKU5 bat-CoV. Whereas 100 % of its E, M and N protein’s antigenic sites are found to be conserved with those in HKU4 and HKU5.

**Conclusion:**

This sharing suggests that in case of pathogenicity MERS-CoV is more closely related to HKU5 bat-CoV than HKU4 bat-CoV. The conserved epitopes indicates their evolutionary relationship and ancestry of pathogenicity.

**Electronic supplementary material:**

The online version of this article (doi:10.1186/s13029-016-0049-7) contains supplementary material, which is available to authorized users.

## Background

Coronavirus, the members of Coronaviridae family are the diverse group of virus which infects domestic animals, birds as well as human. Coronaviruses are enveloped RNA viruses which are classified into four genera, Alpha coronavirus, Beta coronavirus, Gamma coronavirus and Delta coronavirus [1]. HCoV-229E, HCoV-OC43, SARS-CoV, HCoV-NL63, HCoV-HKU1 and MERS-CoV are the six types of human coronaviruses evolved in between 1960 and 2015 whereas MERS-CoV is newly emerged strain. This newly emerged MERS-CoV, which is highly fatal, belongs to lineage C of the genus Beta coronavirus [2]. Human coronaviruses have been tracked down to zoonotic origin. Among the six strains of human corona-viruses, the first HCoV-229E has structural similarity with Bat coronaviruses. This phenomenon resemble to other members that are also have originated from different animal corona-virus like HCoV-OC43 from bovine corona-virus, SARS-CoV and HCoV-NL63 from bat or palm civet corona-virus and HCoV-HKU1 from Mouse hepatitis virus (MHV). Like other human coronaviruses, it is assumed that MERS-CoV has been evolved from zoonotic origin but the zoonotic source of MERS-CoV remains unknown [3–5].

Some studies identified some close amino acid similarity between MERS-CoV and *Nycteris* and *Pipistrellus* bat species [6]. But recent reports identified that MERS-CoV is more closely related to Tylonycteris bat CoV HKU4 (Ty-BatCoV HKU4) and Pipistrellus bat CoV HKU5 (Pi-BatCoV HKU5) [7]. MERS-CoV and Bat-CoV HKU5 bat corona-viruses shared high degree of amino acid similarity in their RNA polymerase (92.1 to 92.3 %), 3C-like protease (82 %), polymerase (92 %), and proofreading exonuclease (91 %) and nucleocapsid (N) protein (68 %) [8, 9]. But it is more closely related to Ty-BatCoV HKU4 in S and N. The major difference between MERS-CoV and these bat corona-viruses is in the region between the spike and the envelop genes. The MERS-CoV has five ORFs while the bat viruses have four in this region [3–5, 10].

Though the MERS-CoV is structurally related to the bat-CoV but there is no report of the sharing of antigenic sites among those corona-viruses. To better understand the evolutionary origin of MERS-CoV pathogenicity it is really needed to know in which extent they are conserved in their immunogenicity.

In this study, we identify the conserved antigenic site among MERS and Bat Corona-virus. For this, bioinformatics analyses of their spike (S), membrane (M), enveloped (E) and nucleocapsid (N) proteins were done for finding the conserved antigenic sites and for mapping the evolutionary conserved antigenic sites on their 3D structures which were determined by threading modeling technique.

## Methods

### Retrieving MERS and Bat coronavirus protein sequences

A total of available five spike (S), membrane (M), enveloped (E) and nucleocapsid (N) protein sequence of HKU4, HKU5 Bat-CoV and 62 S, 64 E, M and 72 N protein sequences of MERS-CoV were retrieved from NCBI GenBank sequence database [11] (Additional file 1: Table S1).

### Identifcation of conserved region

Retrieved sequences were aligned using EBI-clustalW program [12] to find the conserved region. This multiple sequence alignment (MSA) was done with Gonnet matrix [12] and predicted their phylogenetic relationship (Mmaximum Parsimony, MP) by using MEGA 5.0 [13] to understand the conserved regions among them. From the multiple sequence alignment, the highest number of identical and similar amino acid containing region was selected as a conserved region. That selected conserved region was then used for antigenic site prediction.

### Detection of immunogenicity of conserved peptides

Immunogenicity of the conserved peptides was determined by using the B cell epitope prediction tools of The Immune Epitope Database (IEDB) [14]. Among B cell epitope prediction tools of IEDB, Bepipred linear epitope prediction method [15] and Ellipro-structural based discontinuous epitope prediction methods were applied [14]. The antigenic sites of MERS coronavirus spike, envelope, membrane and nucleocapsid proteins were also determined by using Bepipred and Ellipro analysis. Among Bepipred and Ellipro predicted epitopes, fully or at least 90 % overlapping epitopes were chosen as the desired epitopes.

### Prediction of epitope conservancy

To check the conservancy of the predicted epitopes the epitope conservancy analysis tool from the IEDB analysis resource [16] was used. This tool calculates the conservancy level by searching for identities in the given protein sequence.

### Prediction and evaluation protein 3D model

As the experimental structure of spike (S), membrane (M), enveloped (E) and nucleocapsid (N) proteins of any MERS coronavirus isolate were not found in protein data bank (PDB), their 3D structures were predicted by using I-TASSER server [17]. I-TASSER server gives protein 3D structure by multiple threading alignments [17]. I-TASSER provided top models quality were then verified by PROCHECK analysis [18]. The model in which maximum numbers of amino acid residues were found to be in the most favorable region was selected as the best model. This model was then used to locate the epitope by using UCSF Chimera [19] visualization tool.

## Results

### MERS and Bat (HKU4, HKU5) coronaviruses are found to be mostly conserved in case of envelope protein

**Fig. 1:**
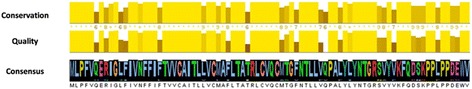
Multiple sequence alignment of MERS and HKU4 coronavirus envelope (E) protein: Multiple sequence alignment of total 64 numbers of MERS-CoV and 5 numbers of HKU4 bat coronaviruse sequences indicates that are highly conserved in envelope (E) protein. Conservation showed here is based on 11 base scales where yellow color bar and star sign indicates the full conservation. Alignment quality was based on BLOSUM 62 substitution matrix score where yellow color indicates good quality. All the colors changes according to the conservation and alignment quality. Black bars showed the consensus sequence. This alignment was visualized by Jalview 2.8 [22] and color scheme used is Clustalx

**Fig. 2:**
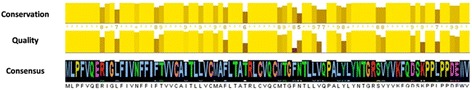
Multiple sequence alignment of MERS and HKU5 coronavirus envelope (E) protein: Figure legend is as in Fig. 1

In case of envelope protein, MERS coronaviruses are found to be highly conserved with HKU4 and HKU5 bat coronaviruses (Figs. 1, 2 respectively) compared to the other proteins (data not shown). From the maximum parsimony phylogenetic analysis of MEGA 5.0, it is found that spike (S), envelope (E), membrane (M) and nucleo-capsid (N) protein MERS-CoV has relationship with Bat (HKU4 and HKU5) coronavirus (Additional file 2: Figure S1, Additional file 3: Figure S2, Additional file 4: Figure S3 and Additional file 5: Figure S4 respectively).

### S, E, M, N protein’s conserved regions are predicted to be antigenic

The MSA derived conserved region were used to determine the antigenic sites by using IEDB resource analysis B cell epitope prediction tool [14]. From this analysis, a total of 3 epitope from S protein, 1 epitope from E protein, 4 epitope from M protein and 5 epitope from N protein were found from the HKU4 bat and MERS coronavirus conserved region (Table 1). Similarly, 7 epitope from S protein, 1 epitope from E protein, 4 epitope from M protein and 5 epitope from N protein were found from the HKU5 bat and MERS coronavirus conserved region (Table 2).

**Table 1:** Predicted antigenic sites, their lengths and their conservancy using IEDB [14] analysis tool from MERS and HKU4 Bat coronavirus conserved protein region

Protein	Peptide	Length (aa)	Identity (%)
Spike (S)	LLSGTPPQVY	10	92.54
	IADPGYMQG	9	100.00
	DAVNNNAQ	8	92.54
Envelope (E)	DSKPPLPPDEWV	12	92.75
Membrane (M)	WSFNPE	6	100.00
	DRLPNEV	7	92.75
	SYGTNS	6	92.75
	AGNYRSPPIT	10	92.75
Nucleo-capsid (N)	DRKINT	6	100
	TGPEAAL	9	93.51
	LRGPGDLQGN	10	93.51
	TEDPRWPQI	9	93.51
	HQNNDDHGN	9	93.51

**Table 2:** Predicted antigenic sites, their lengths and their conservancy using IEDB [14] analysis tool from MERS and HKU5 Bat coronavirus conserved protein region

Protein	Peptide	Length (aa)	Identity (%)
Spike (S)	SQYSRS	6	92.54
	KSSQSSPIIPGFG	13	92.54
	SISTGSRSARS	11	89.55
	IADPGYMQG	9	100.00
	DAVNNNAQ	8	92.54
	IQSDRK	6	92.54
	LLSGTPPQVY	10	92.54
Envelope (E)	DSKPPLPPDEWV	12	97.25
Membrane (M)	WSFNPE	6	100.00
	DRLPNEV	7	92.75
	SYGTNS	6	92.75
	AGNYRSPPIT	10	92.75
Nucleocapsid (N)	DRKINT	6	100.00
	TGPEAAL	7	94.74
	LRGPGDLQGN	10	94.74
	TEDPRWPQI	9	100.00
	HQNNDDHGN	9	94.74

### One epitope of S, M and N protein is fully conserved among MERS and Bat coronavirus

The conservancies of all epitopes were determined by IEDB conservancy analysis tools [16]. Among the IEDB predicted epitopes, most of the epitopes are found to be >90 % conserved among MERS and Bat (HKU4, HKU5) coronaviruses (Tables 1, 2). Among these epitopes, one epitope of S, M, N proteins are found to be 100 % conserved.

### MERS and Bat coronaviruses shared common B cell epitopes

**Fig. 3:**
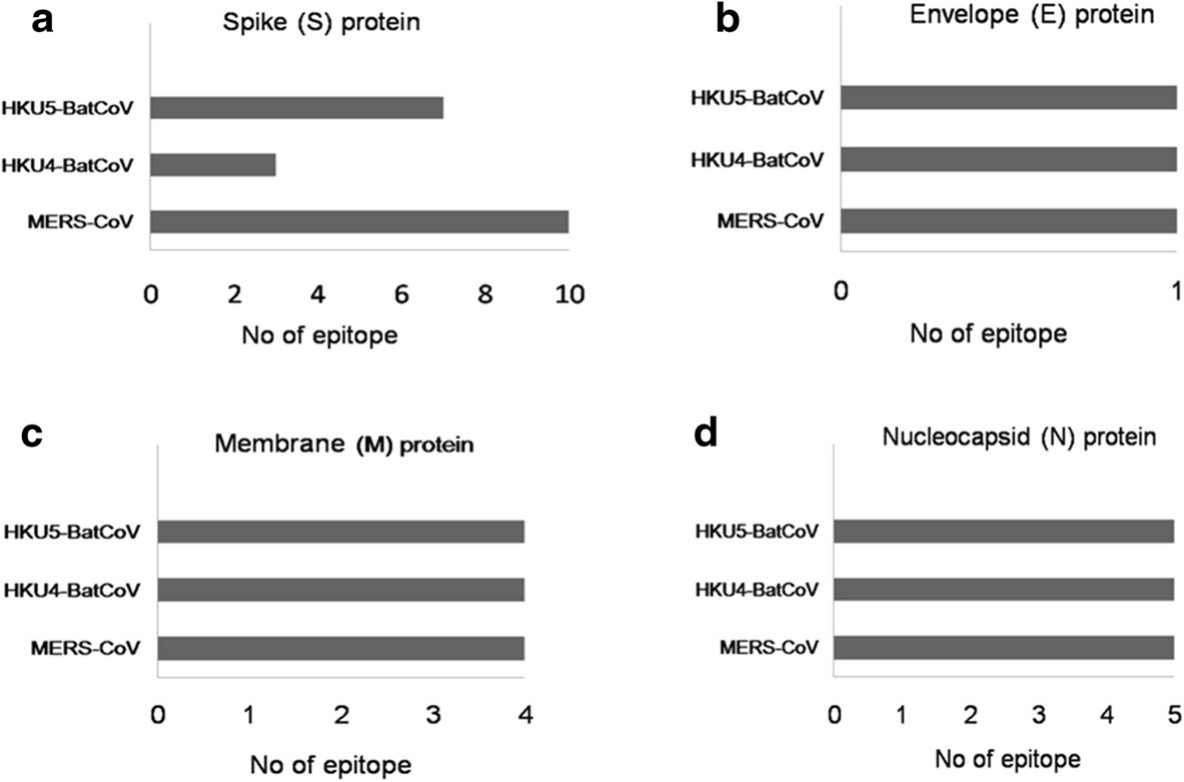
MERS-CoV shared S, E, M and N proteins epitope with HKU4 and HKU5 bat coronavirus: **a** MERS-CoV shared maximum number of spike protein epitope with HKU5 bat- CoV than HKU4 Bat-CoV. Here Y axis indicates the coronavirus strain and X axis indicates the epitopes. **b** MERS-CoV shared equal number of envelope protein epitope with HKU4 and HKU5 bat-CoV. **c** In case of membrane protein epitope, they shared equal number of epitope. **d** MERS and Bat coronaviruses shared equal number of nucleocapsid protein epitope

From the IEDB predicted epitopes of MERS coronavirus S, E, M and N proteins (Table 3), it is found that most of the epitopes are common between MERS and Bat coronavirus. They shared approximately 100 % of E, M and N proteins epitope. In case of S protein, HKU5 shared around 70 % epitope with MERS-CoV while HKU4 shared only 30 % epitope (Fig. 3).

**Table 3:** MERS coronavirus spike, envelop, membrane and nucleocapsid proteins antigenic sites predicted by IEDB analysis [14]

Protein	Peptide	Length (aa)
Spike (S)	GNFSDG	6
	IQSDRK	6
	SYTGSSFYAPEPITS	15
	QYGTDTNSV	9
	SQYSRS	6
	KSSQSSPIIPGFG	13
	SISTGSRSARS	11
	IADPGYMQG	9
	DAVNNNAQ	8
	LLSGTPPQVY	10
Envelope (E)	DSKPPLPPDEWV	12
Membrane (M)	WSFNPE	6
	DRLPNEV	7
	SYGTNS	6
	AGNYRSPPIT	10
Nucleocapsid (N)	DRKINT	6
	TGPEAAL	7
	LRGPGDLQGN	10
	TEDPRWPQI	9
	HQNNDDHGN	9

### A tertiary structure of S, E, M, N proteins was predicted and validated using in silico approach

**Fig. 4:**
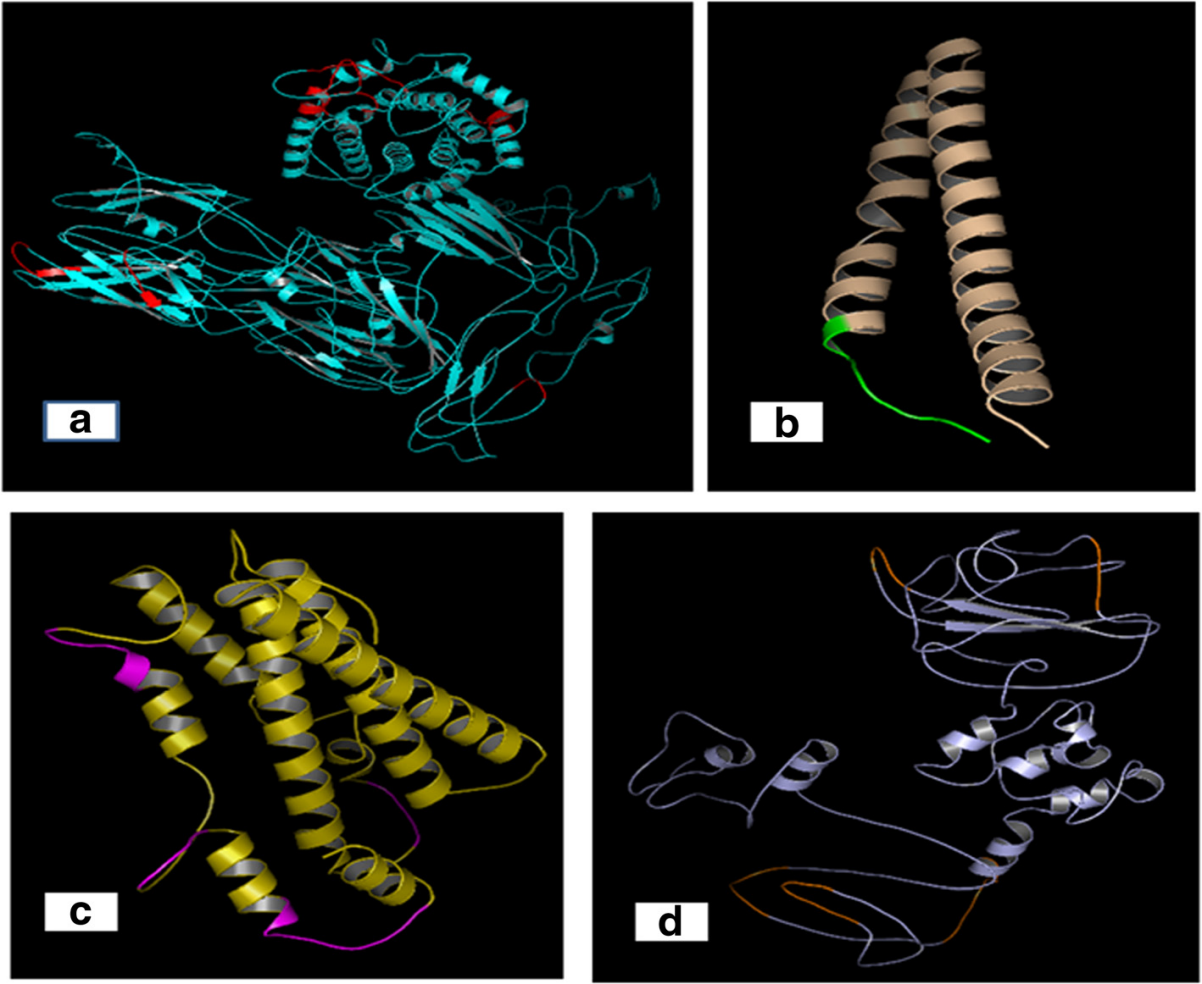
3D structure of MERS-CoV S, E, M and N protein: **a** Spike (S) protein: Predicted conserved S protein epitopes are mapped onto protein 3D structure using UCSF Chimera [19] visualization tool. Each epitopes are labelled with red color. **b** Envelope (E) protein: Figure legend as Fig. 4(a). Epitopes are marked as green color. **c** Membrane (M) protein: Figure legend as Fig. 4(a). Epitopes are labelled with magenta color. **d** Nucleo-capsid (N) protein: Figure legend as Fig. 4(a). Conserved epitopes are labeled with orange color

As the experimental tertiary structure of the S, E, M, N proteins is not available, we modeled a 3D structure by I-TASSER server [17] by multiple threading alignments. I-TASSER analysis deduced 5 different models (data not shown) for this protein. The quality of prediction of all the protein models was checked by PROCHECK analysis [18]. The model in which maximum numbers of amino acid residues were found to be in the most favorable region was selected as the best model. Using UCSF Chimera visualization tool [19], all the conserved (>90 %) epitopes are mapped on the predicted S, E, M and N protein structures (Fig. 4).

## Discussion

Coronaviruses are the most diverse groups of virus which have emerged as deadly viruses in course of time. Most of the human coronaviruses are evolved from zoonotic origin. In most cases bats are served as a reservoir for zoonotic viruses [20]. SARS-CoV has originated from animals, with horseshoe bats as the natural reservoir and palm civet as the intermediate host allowing animal to-human transmission. The HCoV-229E has structural similarity with Bat corona-viruses [21]. Similarly SARS-CoV, HCoV-229E, HCoV-NL63 have originated from the bat but the zoonotic source of MERS-CoV is still not clear [3]. Though the MERS-CoV is found to be structurally related to the bat corona-viruses (HKU4 and HKU5) but there is no report of the sharing of antigenic sites among them. To better understand the evolutionary origin of MERS-CoV pathogenicity we need to know in which extent they are conserved in their immunogenicity.

To address pathogeneic relationship, we have constructed a phylogenetic tree and analyzed the relationship of MERS and Bat coronaviruses using the spike (S), envelope (E), membrane (M), nucleocapsid (N) proteins sequences. It is found that MERS-CoV has phylogenetic relationship with HKU4 and HKU5 bat-CoV. We also predicted conserved antigenic sites and found that, MERS and HKU4 bat corona-viruses shared 30 % of S protein epitope and 100 % of E, M and N proteins epitope. And MERS and HKU5 bat coronaviruses shared 70 % of S protein epitope and 100 % of E, M and N proteins epitope. In most cases conservation level found >90 %. These findings suggested that, in case of antigenicity MERS-CoV is more closely related to HKU5 bat-CoV than the HKU4 bat-CoV. This study showed how pathogenically HKU4 and HKU5 bat-CoVare closely related to the MERS-CoV. Therefore, the level of conservation among antigenic sites provides evidence in support of their ancestry of pathogenicity.

## Conclusions

This study reveals that MERS and Bat coronaviruses shared some common antigenic sites in their spike (S), envelope (E), membrane (M) and nucleo-capsid (N) protein. The shared epitopes are over 90 % conserved throughout their evolutionary process. This shared epitopes also show that, in case of antigenic sites, MERS-CoV is more closely related to HKU5 bat coronaviruses than HKU4 bat coronaviruses. The conserved antigenic sites strongly support their ancestry relationships.

## Additional files

Additional file 1: Table S1. Sequence related information. (XLSX 12 kb) Additional file 2: Figure S1. Phylogenetic analysis of MERS and Bat (HKU4 and HKU5) coronavirus S protein: The evolutionary history was inferred using the Maximum Parsimony method. Tree #1 out of 5 most parsimonious trees (length = 3378) is shown. The consistency index is 0.990823 (0.990823), the retention index is 0.996655 (0.996655), and the composite index is 0.987508 (0.987508) for all sites and parsimony-informative sites (in parentheses). The MP tree was obtained using the Subtree-Pruning-Regrafting (SPR) algorithm with search level 0 in which the initial trees were obtained by the random addition of sequences (10 replicates). The analysis involved 72 amino acid sequences. All positions containing gaps and missing data were eliminated. There were a total of 1347 positions in the final dataset. Evolutionary analyses were conducted in MEGA5 [13]. (TIF 328 kb) Additional file 3: Figure S2. Phylogenetic analysis of MERS and Bat (HKU4 and HKU5) coronavirus E protein: The evolutionary history was inferred using the Maximum Parsimony method. Tree #1 out of 10 most parsimonious trees (length = 40) is shown. The consistency index is 1.000000 (1.000000), the retention index is 1.000000 (1.000000), and the composite index is 1.000000 (1.000000) for all sites and parsimony-informative sites (in parentheses). The MP tree was obtained using the Subtree-Pruning-Regrafting (SPR) algorithm with search level 0 in which the initial trees were obtained by the random addition of sequences (10 replicates). The analysis involved 74 amino acid sequences. All positions containing gaps and missing data were eliminated. There were a total of 82 positions in the final dataset. Evolutionary analyses were conducted in MEGA5 [13]. (TIF 360 kb) Additional file 4: Figure S3. Phylogenetic analysis of MERS and Bat (HKU4 and HKU5) coronavirus M protein: The evolutionary history was inferred using the Maximum Parsimony method. Tree #1 out of 2 most parsimonious trees (length = 312) is shown. The consistency index is 0.990385 (0.990033), the retention index is 0.995940 (0.995940), and the composite index is 0.986364 (0.986014) for all sites and parsimony-informative sites (in parentheses). The MP tree was obtained using the Subtree-Pruning-Regrafting (SPR) algorithm with search level 0 in which the initial trees were obtained by the random addition of sequences (10 replicates). The analysis involved 74 amino acid sequences. All positions containing gaps and missing data were eliminated. There were a total of 154 positions in the final dataset. Evolutionary analyses were conducted in MEGA5 [13]. (TIF 352 kb) Additional file 5: Figure S4. Phylogenetic analysis of MERS and Bat (HKU4 and HKU5) coronavirus N protein: The evolutionary history was inferred using the Maximum Parsimony method. Tree #1 out of 9 most parsimonious trees (length = 590) is shown. The consistency index is 0.996610 (0.996599), the retention index is 0.999179 (0.999179), and the composite index is 0.995792 (0.995780) for all sites and parsimony-informative sites (in parentheses). The MP tree was obtained using the Subtree-Pruning-Regrafting (SPR) algorithm with search level 0 in which the initial trees were obtained by the random addition of sequences (10 replicates). The analysis involved 82 amino acid sequences. All positions containing gaps and missing data were eliminated. There were a total of 411 positions in the final dataset. Evolutionary analyses were conducted in MEGA5 [13]. (TIF 391 kb)
