# Supplementary figures and images for: Conserved antigenic sites between MERS-CoV and Bat-coronavirus are revealed through sequence analysis
Source: Source Code Biol Med. 2016 Mar 9;11:3. doi: 10.1186/s13029-016-0049-7 (PMC4784407; doi:10.1186/s13029-016-0049-7)

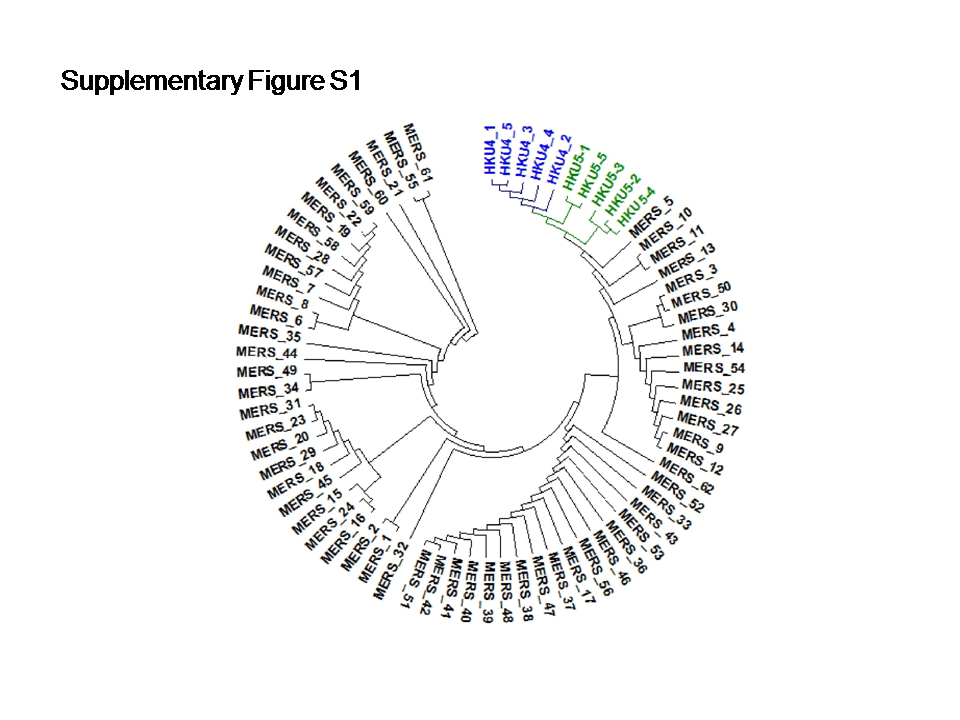

Supplement: Additional file 2: Figure S1. — Phylogenetic analysis of MERS and Bat (HKU4 and HKU5) coronavirus S protein: The evolutionary history was inferred using the Maximum Parsimony method. Tree #1 out of 5 most parsimonious trees (length = 3378) is shown. The consistency index is 0.990823 (0.990823), the retention index is 0.996655 (0.996655), and the composite index is 0.987508 (0.987508) for all sites and parsimony-informative sites (in parentheses). The MP tree was obtained using the Subtree-Pruning-Regrafting (SPR) algorithm with search level 0 in which the initial trees were obtained by the random addition of sequences (10 replicates). The analysis involved 72 amino acid sequences. All positions containing gaps and missing data were eliminated. There were a total of 1347 positions in the final dataset. Evolutionary analyses were conducted in MEGA5 [13]. (TIF 328 kb) [file 13029_2016_49_MOESM2_ESM.tif]

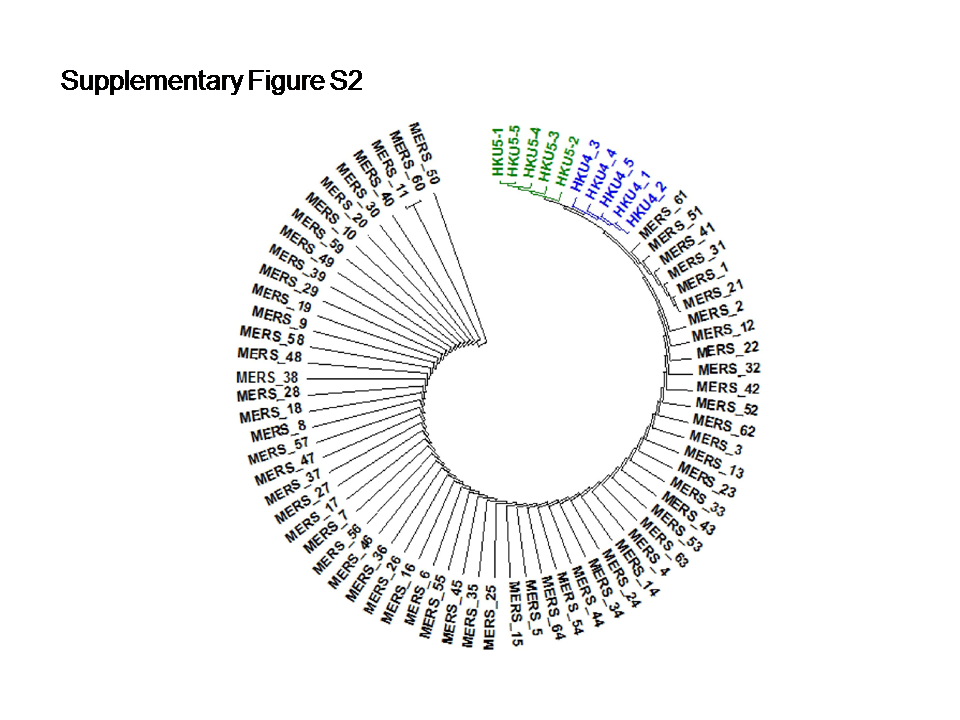

Supplement: Additional file 3: Figure S2. — Phylogenetic analysis of MERS and Bat (HKU4 and HKU5) coronavirus E protein: The evolutionary history was inferred using the Maximum Parsimony method. Tree #1 out of 10 most parsimonious trees (length = 40) is shown. The consistency index is 1.000000 (1.000000), the retention index is 1.000000 (1.000000), and the composite index is 1.000000 (1.000000) for all sites and parsimony-informative sites (in parentheses). The MP tree was obtained using the Subtree-Pruning-Regrafting (SPR) algorithm with search level 0 in which the initial trees were obtained by the random addition of sequences (10 replicates). The analysis involved 74 amino acid sequences. All positions containing gaps and missing data were eliminated. There were a total of 82 positions in the final dataset. Evolutionary analyses were conducted in MEGA5 [13]. (TIF 360 kb) [file 13029_2016_49_MOESM3_ESM.tif]

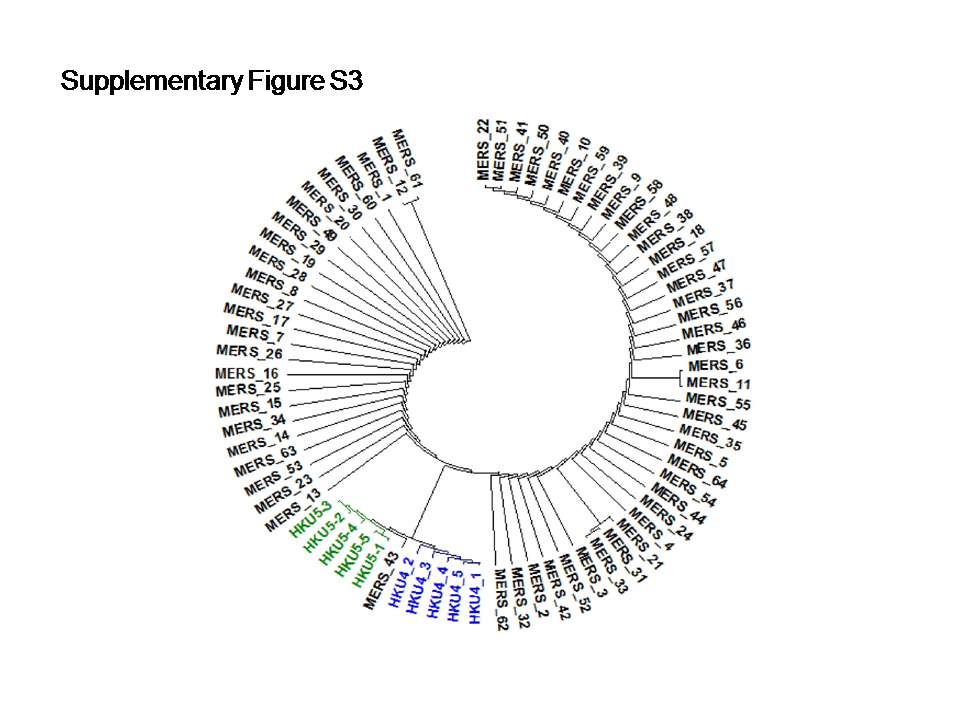

Supplement: Additional file 4: Figure S3. — Phylogenetic analysis of MERS and Bat (HKU4 and HKU5) coronavirus M protein: The evolutionary history was inferred using the Maximum Parsimony method. Tree #1 out of 2 most parsimonious trees (length = 312) is shown. The consistency index is 0.990385 (0.990033), the retention index is 0.995940 (0.995940), and the composite index is 0.986364 (0.986014) for all sites and parsimony-informative sites (in parentheses). The MP tree was obtained using the Subtree-Pruning-Regrafting (SPR) algorithm with search level 0 in which the initial trees were obtained by the random addition of sequences (10 replicates). The analysis involved 74 amino acid sequences. All positions containing gaps and missing data were eliminated. There were a total of 154 positions in the final dataset. Evolutionary analyses were conducted in MEGA5 [13]. (TIF 352 kb) [file 13029_2016_49_MOESM4_ESM.tif]

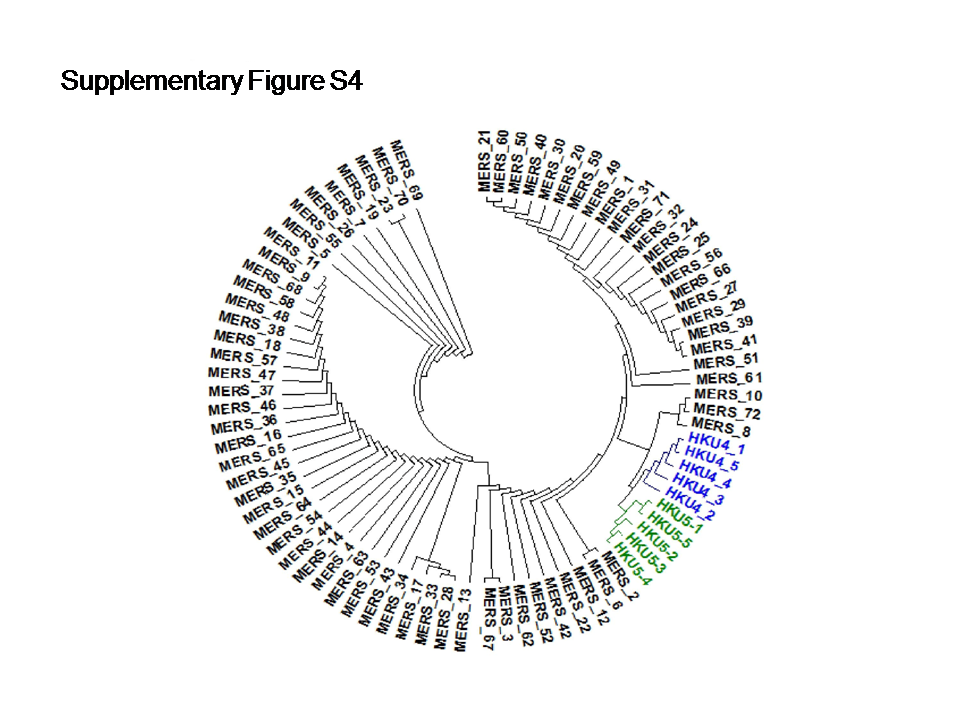

Supplement: Additional file 5: Figure S4. — Phylogenetic analysis of MERS and Bat (HKU4 and HKU5) coronavirus N protein: The evolutionary history was inferred using the Maximum Parsimony method. Tree #1 out of 9 most parsimonious trees (length = 590) is shown. The consistency index is 0.996610 (0.996599), the retention index is 0.999179 (0.999179), and the composite index is 0.995792 (0.995780) for all sites and parsimony-informative sites (in parentheses). The MP tree was obtained using the Subtree-Pruning-Regrafting (SPR) algorithm with search level 0 in which the initial trees were obtained by the random addition of sequences (10 replicates). The analysis involved 82 amino acid sequences. All positions containing gaps and missing data were eliminated. There were a total of 411 positions in the final dataset. Evolutionary analyses were conducted in MEGA5 [13]. (TIF 391 kb) [file 13029_2016_49_MOESM5_ESM.tif]
